# Prediction of pathological complete response and prognosis in patients with neoadjuvant treatment for triple-negative breast cancer

**DOI:** 10.1186/s12885-018-4925-1

**Published:** 2018-10-29

**Authors:** Paul Gass, Michael P. Lux, Claudia Rauh, Alexander Hein, Mayada R. Bani, Cornelia Fiessler, Arndt Hartmann, Lothar Häberle, Jutta Pretscher, Ramona Erber, David L. Wachter, Rüdiger Schulz-Wendtland, Matthias W. Beckmann, Peter A. Fasching, Marius Wunderle

**Affiliations:** 1 Department of Gynecology and Obstetrics, Erlangen University Hospital, University Breast Center for Franconia, Comprehensive Cancer Center Erlangen-EMN, Friedrich Alexander University of Erlangen–Nuremberg, Universitätsstrasse 21–23, 91054 Erlangen, Germany; 2 Department of Medical Informatics, Biometry and Epidemiology, Friedrich Alexander University of Erlangen-Nuremberg, Waldstrasse 6, 91054 Erlangen, Germany; 3 Institute of Pathology, Erlangen University Hospital, Friedrich Alexander University of Erlangen–Nuremberg, Krankenhausstrasse 8–10, 91054 Erlangen, Germany; 4 Biostatistics Unit, Department of Gynecology and Obstetrics, Erlangen University Hospital, Universitätsstrasse 21–23, 91054 Erlangen, Germany; 5 Institute of Diagnostic Radiology, Erlangen University Hospital, Friedrich Alexander University of Erlangen–Nuremberg, Maximiliansplatz 3, 91054 Erlangen, Germany

**Keywords:** Triple-negative breast cancer, Neoadjuvant therapy, Platinum, Pathological complete response, Prognosis, Prediction

## Abstract

**Background:**

It has been reported that pathological complete response is an important surrogate marker for disease-free survival and overall survival in patients with triple-negative breast cancer. This study investigates predictors of the response to neoadjuvant platinum-based or anthracycline-based treatment, and of the prognosis, in patients with triple-negative breast cancer.

**Methods:**

A total of 121 patients with triple-negative breast cancer received neoadjuvant treatment with either platinum or anthracycline between 2008 and 2013. Pathological complete response was assessed relative to different treatments using logistic regression models with age, clinical tumor stage, grading, and Ki-67 as predictors and interaction terms, to obtain adjusted and subgroup-specific results. The impact of the pathological complete response rate on disease-free survival and overall survival was also analyzed.

**Results:**

The pathological complete response rate was higher after platinum/taxane treatment compared with anthracycline/taxane (50.0% vs. 41.8%), but this was not significant in the adjusted analysis (OR 1.44; 95% CI, 0.68 to 3.09). A high histological grade (G3) was a predictor for higher pathological complete response in platinum-based therapy (OR 2.27; 95% CI, 1.00 to 5.30). The effect of neoadjuvant chemotherapy on pathological complete response was significantly different for G1–2 vs. G3 (*P*_interaction_ = 0.013), and additional subgroup-specific differences were noted. Pathological complete response was a predictor for improved disease-free survival and overall survival in both treatment groups, with and without platinum chemotherapy.

**Conclusions:**

This retrospective study of patients with triple-negative breast cancer adds to the evidence that the treatment effect of platinum may be greatest particularly in G3 tumors. In addition, the effect of pathological complete response on the prognosis does not depend on the treatment used.

## Background

Breast cancer (BC) is the most frequent type of cancer in women, with approximately 70,000 new cases in Germany per year [1]. There has been substantial improvement in the prognosis and treatment of BC; the prognosis depends mainly on the clinical stage, as well as the molecular characteristics of the tumor [2–8]. The international treatment guidelines focus on stage and on these molecular subtypes, which are characterized immunohistochemically as surrogate markers in clinical routine work [8–10]. Basal-like tumors are very similar to immunohistochemically defined triple-negative breast cancer (TNBC), which lacks expression of the estrogen receptor (ER), progesterone receptor (PR), and human epidermal growth factor receptor 2 (HER2) [11–16].

Systemic treatment for TNBC has been restricted mainly to conventional chemotherapy in the past [8–10, 14, 17], but new targeted therapies such as immune-checkpoint inhibitors [18] and poly-adenosine diphosphate ribose polymerase (PARP) inhibitors [19, 20] have been under investigation in clinical trials, which are suitable for subgroups of TNBC patients with a germline *BRCA1/2* mutation or a homologous recombination deficiency (HRD) in the tumor [7, 14, 21, 22]. Interestingly, TNBC is associated with an increased deoxyribonucleic acid (DNA)-repair defect in the tumor cells, which is caused either by germline mutations in genes such as *BRCA1/2, PALB2,* and others [23, 24] or by a somatic HRD, which can be exploited by systemic therapies [21].

As systemic therapies are limited in patients with TNBC, achieving a pathological complete remission (pCR) continues to be important for a favorable long-term prognosis in these patients, as several studies have reported [7, 25–27]. Higher pCR rates have also been described for TNBC in comparison with other types of BC, especially with platinum treatment [14, 27–36].

Platinum induces DNA damage by cross-linking DNA strands, which leads to cessation of replication and apoptosis of the tumor cell [37–39]. Since TNBC tumors have a limited DNA repair capacity in comparison with other BC subtypes, platinum appears to be an optimal candidate for achieving a high response rate in these tumors [10, 30, 37, 39–42]. However, there is still ongoing debate on whether platinum is the best treatment for different kinds of TNBC or whether an anthracycline-based treatment should be regularly given instead, as recommended in several national and international guidelines [8, 10, 14].

In the GeparSixto trial (NCT01426880) investigating the effect of adding platinum to doxorubicin/paclitaxel in patients with TNBC and HER2-positive BC, there was a significant increase in the pCR rate in TNBC, from 36.9 to 53.2%, with the addition of platinum (OR 1.94; 95% CI, 1.24 to 3.04; *P* = 0.005), while there was no significant effect in HER2-positive BC, at 36.8% vs. 32.8% with the addition of platinum (OR 0.841; 95% CI, 0.511 to 1.39; *P* = 0.581) [35]. Another subgroup analysis from the GeparSixto trial demonstrated an increased pCR in TNBC and HER2-positive BC after platinum treatment in tumors with a histological grade 3 [35]. A survival analysis for TNBC patients from the same trial showed a longer 3-year disease-free survival (DFS) rate of 85.8% vs. 76.1% for platinum treatment (log-rank test, *P* = 0.0325) [31]. These findings prompted us to perform the present retrospective analysis and to investigate the influence of molecular and clinicopathological predictors on pCR during platinum vs. anthracycline treatment in patients with TNBC, as well as the impact of the tumor response on survival in these patients.

## Methods

### Patient selection

Patients selected for this study were treated for invasive TNBC with neoadjuvant chemotherapy at the University Breast Center of Franconia at Erlangen University Hospital between 2008 and 2013. During this time, a total of 2868 patients were treated for invasive BC at the hospital. Patients without TNBC (*n* = 2546) were excluded, as were patients who did not receive neoadjuvant chemotherapy (*n* = 191) or who did not receive combination therapy with at least platinum/taxane or anthracycline/taxane (*n* = 10). The final group consisted of 121 patients with unilateral tumors.

### Clinical data

All characteristics of the patients and tumors were documented as part of the certification processes required by the German Cancer Society (*Deutsche Krebsgesellschaft*) and by the German Society for Breast Diseases (*Deutsche Gesellschaft für Senologie e.V.*) [43, 44]. For certification, tumor characteristics, treatment data, histopathological characteristics, tumor treatments, and follow-up data have to be documented and are audited annually for each preceding year.

### Pathological data on molecular subtypes

All of the histopathological information used in the analysis was directly documented from the original pathology reports, which were reviewed by two investigators. Tumor grade, ER status, PR status, HER2, and Ki-67 proliferation status were assessed as follows. Grading was determined in accordance with the method described by Elston and Ellis [45]. Monoclonal mouse antibody against estrogen receptor-alpha (clone 1D5, 1: 200 dilution, DAKO, Denmark), monoclonal mouse antibody against the progesterone receptor (clone pgR636, 1: 200 dilution, DAKO, Denmark), and monoclonal antibody against Ki-67 (clone MIB-1, 1: 200 dilution, DAKO, Denmark) were stained using an automated immunostainer (Ventana Ultra) on the pretreatment core biopsies. The percentage and intensity of positively stained cells was included in the pathology reports. The cut-off point for high proliferation determined by Ki-67 staining was regarded as more than 35% positively stained cells, in accordance with a biological analysis presented previously. This cut-off value of Ki-67 was chosen because it distinguished the response to chemotherapy best in TNBC [46]. A polyclonal antibody against HER2 (1: 200 dilution, DAKO, Denmark) was used, and HER2 status was given in the pathology reports as negative, 0, 1+, 2+, or 3+ in accordance with the published guidelines [47]. Tumors with a score of 0 or 1+ were regarded as HER2-negative, and those with a score of 3+ were regarded as HER2-positive. Tumors with a 2+ staining score were tested for gene copy numbers of HER2 by chromogene in-situ hybridization. Using a kit with two probes of different colors (ZytoDot, 2C SPEC HER2/CEN17, Zyto Vision Ltd., Bremerhaven, Germany), the gene copy numbers of HER2 and centromeres of the corresponding chromosome 17 were retrieved. A HER2/CEN17 ratio of ≥2.0 or the presence of ≥6 HER2 signals in the majority of tumor cells was considered as amplification of HER2. Scoring was carried out in a standardized way by a group of dedicated pathologists in routine surgical pathology. The immunohistochemical evaluation of ER, PR, Ki-67, and HER2 and the chromogene in-situ hybridization of HER2 were quality-controlled in accordance with the pathology laboratory’s accreditation standards (ISO/DIN 17020, *Deutsche Akkreditierungsstelle*) and validated successfully by yearly round-robin tests, as required by the German Cancer Society. On the basis of these staining results, patients with negative or low expression (≤ 10%) for ER or PR and negative for HER2 were classified as having triple-negative breast cancer (TNBC).

### Definition of pCR

The definition of pCR is the complete disappearance of all invasive carcinoma cells in the breast and axillary lymph nodes (ypT0/ypN0), which is assessed pathologically in the resected tissue after neoadjuvant chemotherapy [26].

### Statistical considerations

The characteristics of the patients were described using mean and standard deviation (SD) or frequencies and percentages. Logistic regression modeling with interaction terms was performed to estimate the joint effect of chemotherapy and other predictor variables (age at first diagnosis as a categorical variable: < 45 years vs. 45–54 years vs. > 54 years; tumor size before therapy as a categorical variable: ≤ 2 cm vs. > 2 cm; grading as a categorical variable: 1 and 2 vs. 3; and Ki-67 as a categorical variable: < 36% vs. ≥ 36%) on the pCR. The results are given as odds ratios (ORs) with corresponding 95% confidence intervals (CIs). DFS times and overall survival (OS) times were analyzed using Kaplan–Meier curves and log-rank tests. *P* values were not corrected for multiple testing. All analyses were carried out using the R system for statistical computing (version 3.3.2, 2016; R Core Team, Vienna, Austria).

## Results

In this group of 121 retrospectively selected patients with TNBC, 66 patients received platinum/taxane-based neoadjuvant chemotherapy and 55 patients received anthracycline/taxane-based neoadjuvant chemotherapy.

The patient characteristics in the anthracycline-treated cohort and platinum-treated cohort were comparable. In the groups receiving anthracycline-based and platinum-based treatment, respectively, the patients’ mean age at the time of first diagnosis of BC was 48.3 years (SD 9.6) vs. 50.3 years (SD 12.1); 81.8% vs. 84.8% had histological grade 3; 76.4% vs. 78.8% had Ki-67 ≥ 36%; and 63.6% vs. 59.1% had tumor sizes > 2 cm. The patients’ characteristics are listed in detail in Table 1.

**Table 1:** Patient and tumor characteristics relative to chemotherapy

	Anthracycline/taxane-based	Platinum/taxane-based
	n (%) or mean (SD)	n (%) or mean (SD)
Overall	55	66
Age at first diagnosis	48.3 (9.6)	50.3 (12.1)
Age		
< 45 y	19 (34.5)	21 (31.8)
45–54 y	21 (38.2)	19 (28.8)
> 54 y	15 (27.3)	26 (39.4)
Tumor size		
≤ 2 cm	20 (36.4)	27 (40.9)
> 2 cm	35 (63.6)	39 (59.1)
Grading		
1 or 2	10 (18.2)	10 (15.2)
3	45 (81.8)	56 (84.8)
Ki-67		
< 36%	13 (23.6)	14 (21.2)
≥ 36%	42 (76.4)	52 (78.8)
pCR		
No	32 (58.2)	33 (50.0)
Yes	23 (41.8)	33 (50.0)

*pCR* pathologic complete response, *SD* standard deviation

The pCR rates were 41.8% (*n* = 23) for anthracycline-based treatment and 50% (*n* = 33) for platinum-based treatment (Table 1). As expected, the pCR rate was higher in patients with clinically smaller tumor sizes, histological grade 3, and higher Ki-67 levels. In addition, pCR was higher in patients < 54 years in comparison with patients > 54 years (Table 2).

**Table 2:** Pathological complete response rates relative to patient and tumor characteristics

	pCR (no)	pCR (yes)
	n (%) or mean (SD)	n (%) or mean (SD)
Overall	65	56
Age at first diagnosis	51.2 (11.6)	47.3 (10.0)
Age		
< 45 y	20 (50.0)	20 (50.0)
45–54 y	20 (50.0)	20 (50.0)
> 54 y	25 (61.0)	16 (39.0)
Tumor size		
≤ 2 cm	21 (44.7)	26 (55.3)
> 2 cm	44 (59.5)	30 (40.5)
Grading		
1 or 2	13 (65.0)	7 (35.0)
3	52 (51.5)	49 (48.5)
Ki-67		
< 36%	19 (70.4)	8 (29.6)
≥ 36%	46 (48.9)	48 (51.1)
Chemotherapy		
Anthracycline + taxane	32 (58.2)	23 (41.8)
Platinum + taxane	33 (50.0)	33 (50.0)

*pCR* pathologic complete response, *SD* standard deviation

**Fig. 1:**
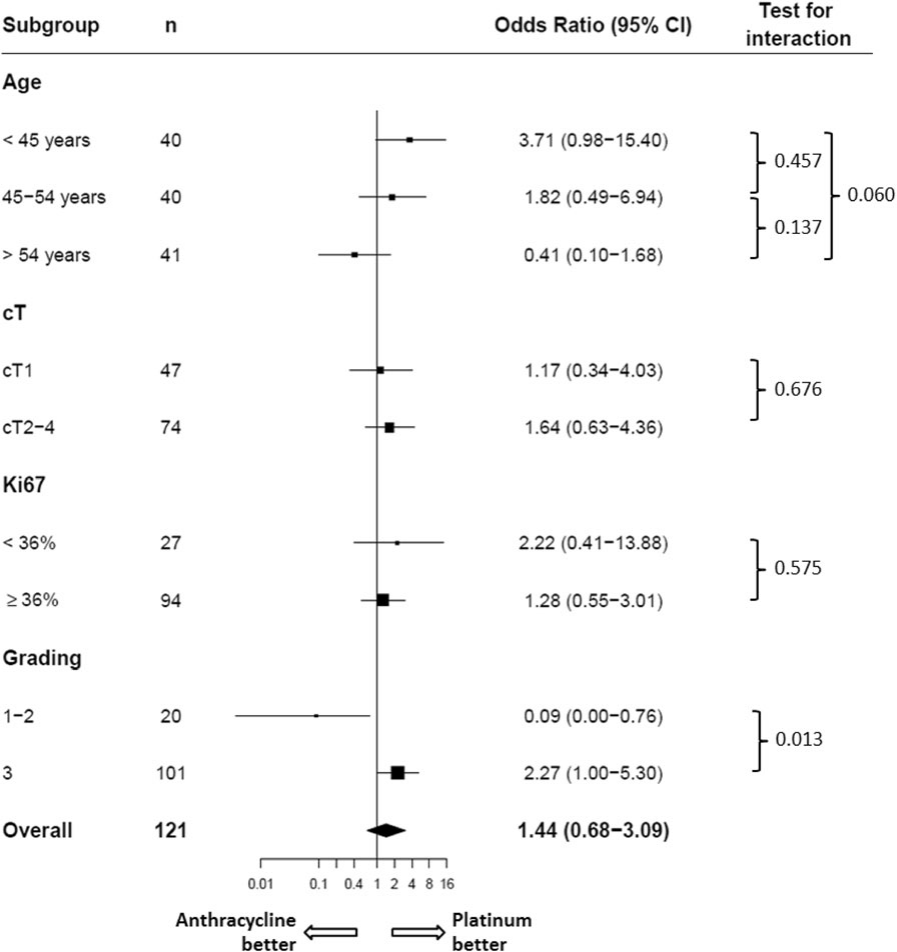
Forest plot for predicting the rate of pathological complete response to treatment in subgroups

For prediction of pCR using a multiple logistic regression model, the OR of platinum treatment vs. anthracycline treatment was 1.44 (95% CI, 0.68 to 3.09). Subgroup analysis for clinical tumor stage, histological grade, and Ki-67 showed a higher pCR rate for grade 3 tumors after platinum treatment (OR 2.27; 95% CI, 1.00 to 5.30). Platinum therapy in grade 1 or grade 2 tumors was associated with an inverse effect on the pCR rate (OR 0.09; 95% CI, 0.00 to 0.76), and the difference in treatment effects for grading (G3 vs. G1/G2) on pCR was statistically significant (*P*_interaction_ = 0.013). The other interactions in the subgroups did not show any statistically significant differences (Fig. 1).

**Fig. 2:**
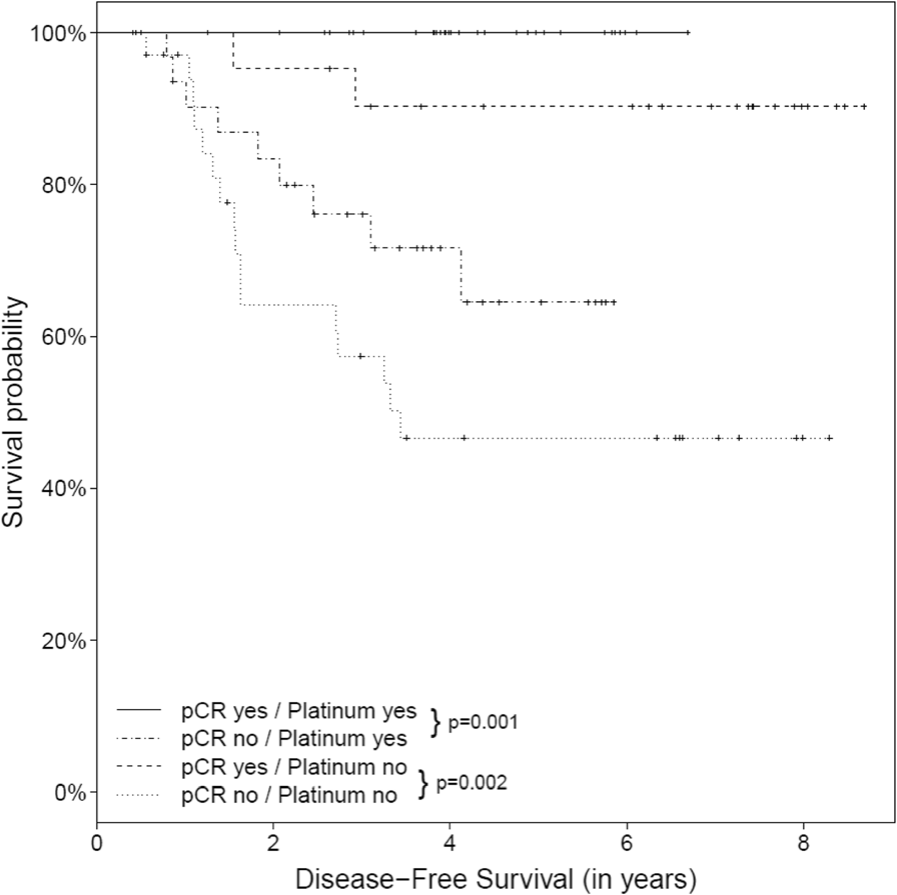
Kaplan–Meier curves for the effect of treatment and pathological complete response (pCR) on disease-free survival

**Fig. 3:**
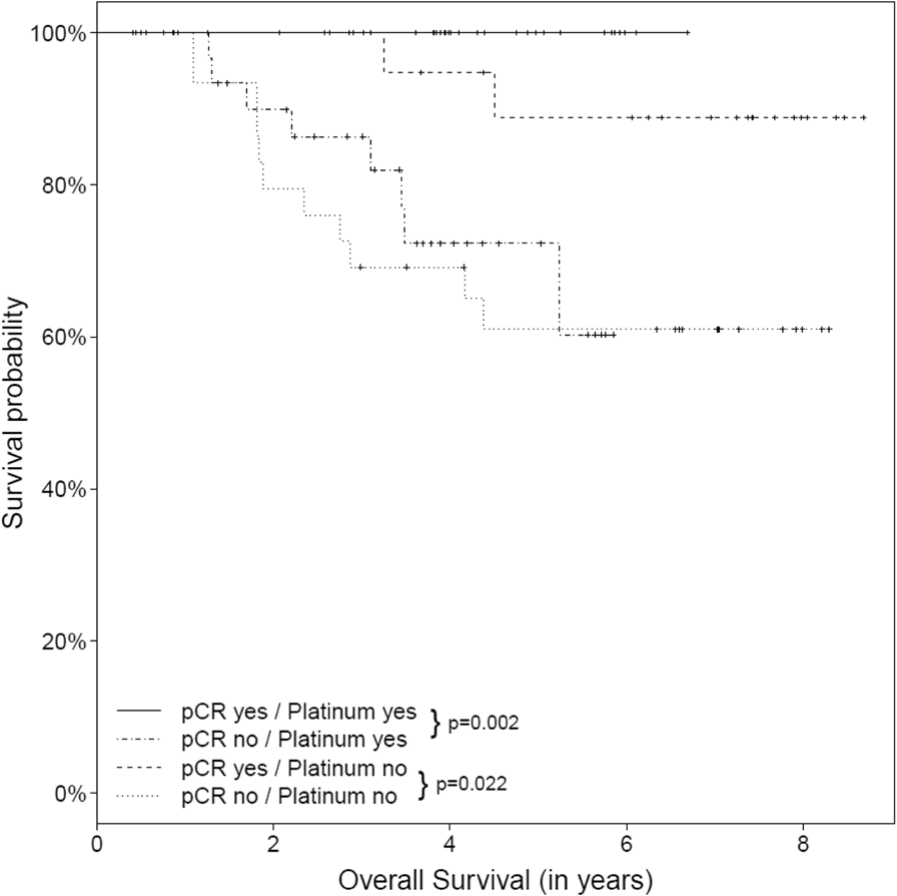
Kaplan–Meier curves for the effect of treatment and pathological complete response (pCR) on overall survival

Whether or not a pCR was achieved was significantly related to increased DFS both in patients who received platinum-based treatment (*P* = 0.001) and in those who received anthracycline-based therapy (*P* = 0.002). The same was seen in relation to OS (*P* = 0.002 for platinum-based therapy and *P* = 0.022 for anthracycline-based therapy) (Figs. 2 and 3).

## Discussion

In this retrospective analysis of neoadjuvantly treated patients with TNBC, no differences were found in the treatment effect of platinum-based chemotherapy and anthracycline-based chemotherapy with regard to pCR for overall TNBC. However, patients with grade 1 or 2 benefited from anthracycline-based chemotherapy whereas patients with grade 3 benefited from platinum-based chemotherapy. Moreover, pCR was a predictor of DFS and OS, independently of the treatment.

Although multivariate analysis showed that the type of chemotherapy was not statistically significant for the prediction of pCR in overall TNBC, there was a trend toward a higher pCR for neoadjuvant platinum-based vs. anthracycline-based therapy. This finding is consistent with several trials that have investigated the effect of different regimens of neoadjuvant chemotherapy in patients with TNBC and have reported higher pCR rates in TNBC after platinum treatment in patients with or without *BRCA1/2* mutations [14, 27–36]. In the present study, histological grade 3 vs. grades 1–2 was a predictor of high effectiveness with regard to platinum treatment in TNBC patients. There is one subgroup analysis from the GeparSixto trial that showed a significant benefit for histological grade 3 vs. grades 1–2 if platinum treatment was added in TNBC and HER2-positive BC (OR for G3, 1.73; 95% CI, 1.15 to 2.60; OR for G1–2, 0.776; 95% CI, 0.432 to 1.40; *P*_interaction_ = 0.027) [35]. A high histological grade may either have an independent influence on the tumor’s sensitivity to platinum treatment or, more likely, it may be a predictor for molecular characteristics of triple-negative tumor cells that predispose to a better response to platinum treatment, such as a higher mutational spectrum, increased HRD [48, 49], faster growth [14, 16], and higher amounts of tumor-infiltrating lymphocytes (TILs) [50, 51].

It has been shown in several publications that *BRCA1/2* mutations, which are associated with increased DNA repair deficiency [20, 38], can be found in at least 10–15% of all TNBCs [24, 32, 52], especially in those with a high grade and Ki-67 [24, 53, 54]. For example, an analysis of 1824 patients with TNBC found a *BRCA1/2* mutation in 4.9% of patients with grades 1–2 and in 13% of patients with grade 3 [24]. These tumors were also associated with a better response to platinum treatment [14, 29, 36, 39].

HRD can be found in tumors with germline or somatic mutations in *BRCA1/2* or in other genes involved in DNA repair [40–42, 48, 49, 55]. In one analysis, the addition of platinum significantly increased the pCR (ypT0is/ypN0) rate from 36.6 to 63.2% in HRD tumors with intact *BRCA1/2 function in tumor tissue (P* = 0.018), marginally from 61.9 to 72.7% in *BRCA*-mutated tumors (*P* = 0.406), and moderately from 20.0 to 40.7% in non-HRD tumors (*P* = 0.086). Patients with HRD-positive tumors had a longer event-free survival than non–HRD-deficient ones (hazard ratio 1.805, *P* = 0.0526) [30]. Other studies support greater efficacy — especially of platinum, but also of anthracyclines — in HRD-positive tumors [40–42, 48]. The same molecular deficiency is targeted by PARP inhibitors. These are under investigation in clinical trials for (neo-) adjuvant treatment of BC, which require either a *BRCA1/2* mutation or a high HRD score for patient inclusion [8, 19, 20, 22]. Stronger efficacy of platinum in high-grade TNBC may be explained by the co-occurrence of high grading and increased HRD, either at the germline level or the somatic level, or both [30, 40, 42, 48], although there is limited data on this issue [48, 49]. In one smaller analysis of 70 TNBC patients, 71% had an increased HRD score. Seventy-six percent of the patients had G3 tumors, 24% had G2 tumors, and no patients with G1 tumors were included in this dataset [48].

It has been shown that Ki-67 is a useful prognostic factor in BC [46, 56], but there are still contradictory data regarding Ki-67 as a predictive factor [57, 58]. In the GeparSixto trial, Ki-67 was not found to have an influence on the tumor response to platinum [35]. In our study, tumors with higher Ki-67 levels were more likely to reach pCR, but the test for interaction did not reveal an association of the Ki-67 level with the prediction of pCR. This implies that in TNBC it is possibly not Ki-67 itself, but other related molecular factors that predict the response to platinum.

Moreover, tumors with poor grading have been reported to show increased amounts of TILs [50, 51, 59]. High stromal TILs (≥ 60%) were found in 27% of G3 tumors, compared with 12% in G2 tumors and 6% in G1 tumors [50]. In TNBC with high TILs, pCR and prognosis were also improved for different treatments [50, 51, 59]. Furthermore, in patients with triple-negative lymphocyte-predominant BC, the addition of platinum treatment to anthracycline treatment improved pCR more (74% vs 43%, *P* = 0.005) than in non–lymphocyte-predominant BC (34% vs. 46%, *P* = 0.08), although the test for interaction was not significant [51].

The present analysis did not investigate the rates of germline mutations, somatic mutations, HRD, or TILs. Nevertheless, when the published data are taken into account, it may be assumed that these factors are more frequently present in the cohort of G3 tumors. This could be the reason for higher platinum sensitivity in tumors with grade 3 and poorer efficacy of platinum in tumors with grades 1–2, in which these factors are less important. Anthracyclines have different mechanisms of action, such as intercalation between base pairs of the DNA/RNA strand, inhibition of the topoisomerase II enzyme, generation of free oxygen radicals, interaction with histones, etc. [60, 61]. It seems that in G1 and G2 tumors, these effects of anthracyclines are more important than those of platinum. Finally, the question arises of whether the total histological score proposed by Elston and Ellis is appropriate for identifying tumors with reduced HRD or other factors of increased chemosensitivity, or whether single criteria such as gland formation, nuclear polymorphism, and mitotic count are more important for distinguishing these tumors [45].

The main weaknesses of the present study are its small sample size and the retrospective nature of the analysis. However, although all the patients were selected from clinical routine work, no unexpected associations were seen. The results of the subgroup analysis must be regarded as mainly exploratory, but the different efficacy relative to grading supports the results of other studies and might require further analysis in the future. Due to the low number of events, multivariate analyses for prediction of DFS and OS were not performed.

## Conclusion

In conclusion, this retrospective study observed higher pCR rates after neoadjuvant therapy with platinum in TNBC patients with G3 tumors. The study indicates that platinum treatment should be considered in the subgroup of high-grade TNBC patients. The results of this study justify further research in larger prospective trials, including the assessment of grading, Ki-67, mutation status, HRD, and TILs in patients with TNBC and possibly other subtypes of BC as well.

## Abbreviations

BC: Breast cancer
CI: Confidence interval
DFS: Disease-free survival
DNA: Deoxyribonucleic acid
ER: Estrogen receptor
HER2: Human epidermal growth factor receptor 2
HRD: Homologous recombination deficiency
OR: Odds ratio
OS: Overall survival
PARP: Poly-adenosine diphosphate ribose polymerase
pCR: Pathological complete response
PR: Progesterone receptor
SD: Standard deviation
TIL: Tumor-infiltrating lymphocyte
TNBC: Triple-negative breast cancer
